# Transactivation Function-1-Mediated Partial Agonist Activity of Selective Estrogen Receptor Modulator Requires Homo-Dimerization of the Estrogen Receptor α Ligand Binding Domain

**DOI:** 10.3390/ijms20153718

**Published:** 2019-07-30

**Authors:** Yukitomo Arao, Kenneth S. Korach

**Affiliations:** Receptor Biology Section, Reproductive and Developmental Biology Laboratory, National Institute of Environmental Health Sciences/NIH, Durham, NC 27709, USA

**Keywords:** estrogen receptor alpha, selective estrogen receptor modulator, homo-dimerization, ligand binding domain, F-domain, AF-1

## Abstract

The isolation of estrogen receptor alpha (ERα) cDNA was successful around 30 years ago. The characteristics of ERα protein have been examined from various aspects, primarily through in vitro cell culture studies, but more recently using in vivo experimental models. There remains, however, some uncharacterized ERα functionalities. In particular, the mechanism of partial agonist activity of selective estrogen receptor modulators (SERMs) that involves control of the N-terminal transcription function of ERα, termed AF-1, is still an unsolved ERα functionality. We review the possible mechanism of SERM-dependent regulation of ERα AF-1-mediated transcriptional activity, which includes the role of helix 12 of ERα ligand binding domain (LBD) for SERM-dependent AF-1 regulation. In addition, we describe a specific portion of the LBD that associates with blocking AF-1 activity with an additional role of the F-domain in mediating SERM activity.

## 1. Introduction

Estrogen receptor alpha (ERα) is the first estrogen receptor protein that the cDNA was isolated from various species, including human, mouse, rat and chicken [1,2,3,4]. Since then, ERα protein has been examined extensively. However, there are some ERα protein functionalities that remain uncharacterized. The molecular mechanism of partial agonist activity of selective estrogen receptor modulators (SERMs), controlling the N-terminal transcriptional activity of ERα is still unsolved. In this review, we describe a cooperative functionality of the ERα protein domains for eliciting a tissue selective gene response by controlling the N-terminal transcriptional activity.

**Figure 1:**
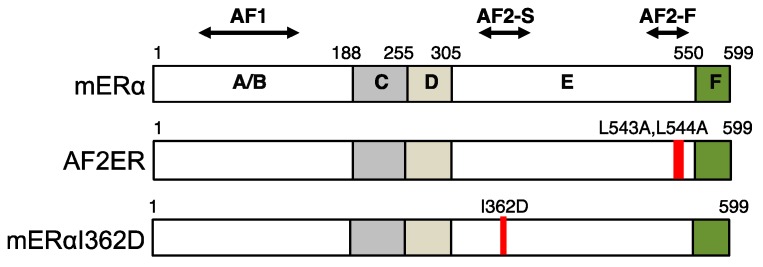
Schematic structure of mouse estrogen receptor α (mERα). ERα consists of six domains named A to F. C-domain binds to an estrogen responsive DNA element. The ligands bind to E-domain (ligand binding domain; LBD). A/B-domain possesses the transactivation function 1 (AF1). E-domain possesses ligand-dependent transcription activation domain (i.e., AF-2). AF-2 is composed of static region (AF2-S) and flexible region (AF2-F). AF2-F corresponds to the helix 12 of LBD. The schematic structures of AF2-F mutant (AF2ER) and AF2-S mutant (mERα-I362D) are shown. Red bars indicate the position of mutated amino acids in the AF-2 mutants.

ERα is a member of the nuclear receptor superfamily that includes the receptors for various lipid-soluble bioactive compounds, such as steroid hormones, vitamin A, vitamin D and bioactive-lipids [5,6,7]. Members of the nuclear receptor superfamily work as ligand-dependent transcription regulators, which possess six highly conserved structural and functional domains termed A through F. As the ligand-dependent transcription regulators, the family members contain a highly conserved DNA binding domain (DBD) also known as the C-domain, and a ligand-binding domain (LBD) referred to as the E-domain. Additionally, each receptor contains more variable domains, termed AB-domains/N-terminal structure, D-domain/hinge region or connective region between DBD and LBD, and F-domain/C-terminal structure [7]. ERα possesses A through F domains and two transactivation functional regions, termed AF-1 and AF-2, which are localized in AB-domains and E-domain, respectively (Figure 1).

The transcriptional activity of AF-2 corresponds to the ligand-dependent transformation of LBD. Especially, the ligand-dependent relocation of the helix 12 of LBD defines the activity of estrogenic chemicals as agonists/ligands that activate transcription or antagonists/ligands that inactivate transcription. We have characterized the specific mutations in helix 12, named AF2ER (i.e., leucines 543 and 544 of mouse ERα were mutated to alanines; L543A, L544A) and found that the antagonists function as agonists through AF2ER mutant ERα in vitro and in vivo [8,9]. A human ERα L540Q mutant, which mutated residue corresponds to the mouse ERα L544, possesses similar characteristics to AF2ER [10]. The first subsection of this review focuses on the functionality of ERα helix 12 for defining the agonistic or antagonistic activities of estrogenic chemicals based on our study using the AF2ER mutant.

The AF-1 is localized to the N-terminus of ERα, which is not involved in the ligand binding. Thus, it has been reported that growth factors may modulate the activity of ERα AF-1-mediated transcription through phosphorylation of specific serine residues in the N-terminus, especially serines 104, 106 and 118 of human ERα. Several in vitro studies have revealed specific protein kinases, such as mitogen-activated protein kinase and cyclin-dependent kinases which are potentially activated by growth factor signaling and induce phosphorylation of these serine residues [11,12,13,14]. In addition, a study using the global ERα knockout (KO) mouse revealed that the growth factors (e.g., Igf1 and Egf)-dependent uterine epithelial cell proliferation was attenuated in the ERα KO mouse [15]; the observation has supported the in vivo biological functionality of ERα AF-1 phosphorylation. Recently, we found that the growth factors failed to induce uterine cell proliferation in the AF2ER mutant mouse, which expresses the ERα protein containing the intact AF-1 and the mutated AF-2 [8]. This result suggested that the proper AF-2 function is necessary for AF-1 activation. Additionally, we identified and reported that a specific region within AF-2 impacts the AF-1 activity [16]. The second subsection of this review describes the possible mechanism of AF-2/LBD-mediated regulation of AF-1 activity.

The length of the F-domain composing the very end of C-terminus is highly variable among the nuclear receptors. For instance, the ERα F-domain contains 50 amino acids that connect directly with helix 12 of the LBD. Because of the high variability in this region among the nuclear receptors, the functionality of F-domain of nuclear receptors is not well understood [17]. We found that the ERα F-domain is involved in the partial agonist activity of SERMs. The third subsection of this review describes the functionality of ERα F-domain for the regulation of SERM-dependent ERα AF-1-mediated transcriptional activity.

SERMs are non-steroidal ER ligands. These chemicals have been generated to treat postmenopausal women for reducing the symptoms of menopause, such as osteoporosis and hot flushes, or for treating breast cancer patients [18,19,20,21,22,23,24,25]. The symptoms of menopause could be normalized by the adjustment of female sex steroid hormone (e.g., 17β-estradiol; E2) level. However, the treatment of E2 to patients affects broader ERα-mediated physiological responses, causing various adverse side effects. The SERMs regulate the activities of ERα AF-1 and AF-2 different from E2. Thus, the treatment with SERMs potentially controls ERα AF-1 or AF-2-mediated physiological responses selectively for reducing side effects. A better understanding of the molecular mechanism of ligand-dependent regulation of ERα AF-1- and AF-2-mediated transcriptional activity related to physiological responses is beneficial for development of more appropriate therapies.

## 2. The Mutation of Helix 12 Reverses Antagonistic Chemicals to Agonists

X-ray crystallography of various nuclear receptors has verified that the overall structure of the LBD is highly conserved among the nuclear receptors. The studies showed that helix 12, the last helix structure of the LBD, is repositioned in a ligand-dependent manner [26]. Specifically, when agonists bind to the ERα LBD, helix 12 cooperates with helices 3 and 4 to create a coactivator interacting surface for transactivation, i.e., AF-2 [27,28]. The receptor interacting motif of coactivators (leucine-x-x-leucine-leucine; LxxLL motif) fits to the cleft of the coactivator interacting surface of the LBD [28]. In contrast, when antagonists/SERMs, such as 4-hydroxytamoxifen (4OHT) bind to the ERα LBD, helix 12 is shifted to the position occupied by the LxxLL motif when agonists bind [29]. It is highly likely that the altered position of helix 12 with antagonists/SERMs prevents coactivator interaction, resulting in attenuation of ERα AF-2-mediated transcriptional activity. When the antagonists/selective estrogen receptor degraders (SERDs), such as fulvestrant/ICI182780 bind to the ERα LBD, helix 12 becomes free from the coactivator interacting surface [30]. This disturbed positioning of helix 12 which is different from that with SERMs is likely to provoke destabilization and proteolysis of the ERα protein [31,32]. Thus, helix 12 would appear to be an important domain for defining the characteristics of different ERα ligands for the ERα transactivation.

We have analyzed the functionality of a specific helix 12 mutated ERα (i.e., mouse ERα L543A-L544A or AF2ER) in vivo and in vitro [8,16,33,34,35]. Originally, Malcolm Parker’s group reported the characteristics of a series of helix 12 mutants, including L543A-L544A and M547A-L548A mouse ERα mutants [36]. The residues of L543, L544, M547 and L548 are located on the same surface of helix 12. Compared to the wild-type ERα, these ERα mutants were reported to exhibit reduced E2-mediated transcriptional activity measured with an estrogen-responsive reporter composed of the consensus estrogen responsive element (ERE) of the vitellogenin (Vit) gene in HeLa cells. In addition, the E2-mediated transcriptional activity was enhanced, rather than inhibited, by antagonists, such as 4OHT or ICI164384 (ICI; the first generation of fulvestrant analogue) [36]. Importantly, the report showed that the effect of antagonist-dependent enhancement was attenuated by truncation of the N-terminal/AB-domains of ERα [36]. They concluded that an intact AF-1 domain is a prerequisite for antagonists-mediated transcriptional stimulation through L543A-L544A and M547A-L548A mutants [36]. The group proposed a possible mechanism for this action. Namely, the SERD (e.g., ICI)-mediated ERα proteolysis was attenuated by these mutations that may reflect the antagonist-dependent transactivation [32,36]. It was insufficient to explain how the AF-1 of mERα L543A-L544A (i.e., AF2ER) is activated by the antagonists.

We observed that the 3x VitERE-mediated transcriptional activity of the AF2ER mutant was intensified in HepG2 cells by various SERMs as well as 4OHT or fulvestrant/ICI182780 [35]. Additionally, we found that E2 was not necessary for antagonist-mediated activation of the AF2ER mutant, which was not addressed in the previous report of Mahfoudi et al [36]. Consistent with the previous study, we found that the AF2ER mutant was not activated by E2, and the antagonist-dependent activation was lost by truncation of the AB-domains of the AF2ER mutant [35]. These results clearly suggested that the AF-1 activity of the AF2ER mutant is dominant for antagonists-mediated transactivation. Further analyses using a mammalian two-hybrid assay (M2H) revealed that the antagonists induced homo-dimerization of AF2ER mutant, which was essential for enhancing transactivation function.

The lack of any ligand-dependent transcription activity of the AF2ER-LBD/EF-domains was an important finding [35]. This allowed us to use the mutant for a M2H assay to examine the homo-dimerization activity of AF2ER-LBD. We found that the antagonist-dependent dimerization activity of AF2ER-LBD was obstructed by a dimerization disruption mutation (leucine 511 of mERα was substituted to arginine, L511R) [37] or by F-domain truncation. Interestingly, the homo-dimerization activity of helix 12 deleted mutant LBD (mERα LBD lacking 542–548 amino acids) was weak but similar to AF2ER-LBD [35]. Importantly, E2 and other agonists did not induce the homo-dimerization activity of AF2ER-LBD. The transcriptional activity of AF2ER mutant through 3x VitERE coincided with the LBD homo-dimerization activity [35]. Furthermore, we revealed that the dimerization disrupted AF2ER mutant (AF2ER-L511R) showed reduced ligand-dependent binding activity to the ERE DNA fragment [35]. These results suggested that the structure of helix 12 is disrupted by the AF2ER mutation and the flanking F-domain influences the antagonist-dependent dimerization of AF2ER-LBD; the dimerized AF2ER mutant can bind an ERE DNA sequence for transcriptional activation. The phenotypes of helix 12 deleted mutant mouse (i.e., mouse ERα lacking 539-554 amino acids or ERαAF2^0^) have been reported as similar to the phenotypes of ERα KO or AF2ER mice [38,39]. Moreover, Movérare-Skrtic et al. reported that fulvestrant works as an agonist in uterus and trabecular bone in ERαAF2^0^ mice [40]. This observation supported our speculation; the destruction of helix 12 structure reverses the function of ERα antagonists. It is likely that the reversed antagonist activity occurs through the antagonist induced homo-dimerization of AF2ER mutant (i.e., helix 12 structure eliminated mutant) on the ERE which then allows exposure of the AF-1 domain for transcriptional activation.

## 3. The Ligand-Dependent Regulation of ERα AF-1 Activity

**Figure 2:**
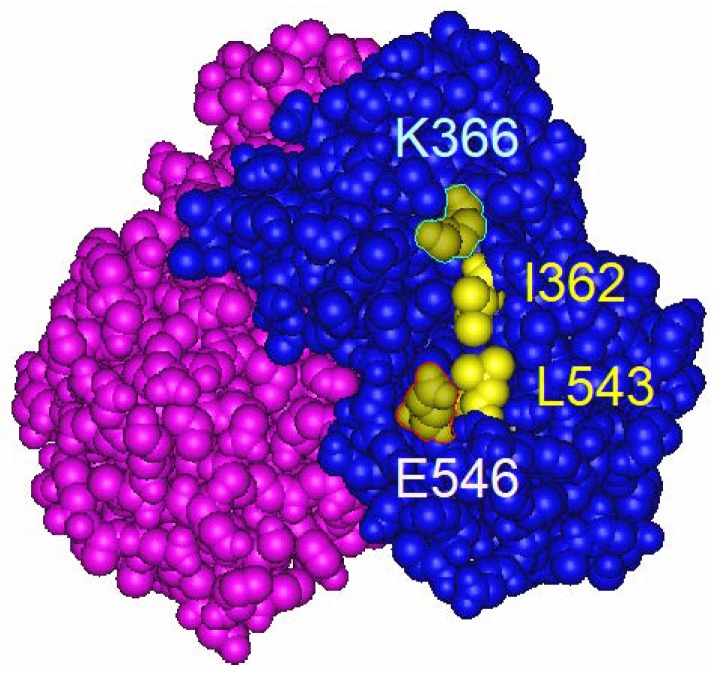
Crystallographic structure of E2-bound LBD dimer of ERα. The diagram was generated from the human ERα LBD with 17β-E2 (Protein Data Bank ID: 1ERE). The dimer form of pink and blue ERα LBD molecules is shown. The residues suggest the position corresponding to the I362, K366, L543 and E546 of mouse ERα. I362 and L543 are hydrophobic amino acids (yellow), K366 is a basic amino acid (blue) E546 is an acidic amino acid (red).

When the agonists bind to the ERα LBD, the flexible helix 12 (AF2-F) and the rigid helices 3 and 4 (AF2-S) form the transcription coactivator binding surface (Figure 1). In the structure of the agonist-bound hERα LBD complexed with the peptide of GRIP1 NR box II (i.e., LxxLL motif containing domain of SRC2), the corresponding residues of mERα lysine 366 (K366) and glutamic acid 546 (E546) were shown to form hydrogen bonds with the LxxLL peptide [29]. It has previously been shown that the position of isoleucine 362 (I362) of mERα, which is localized on the helix 3, is adjacent to the L543 when E2 bound to the LBD (Figure 2). Mak et al. reported that the E2-dependent SRC1e-mediated coactivation function was strongly attenuated by the aspartic acid substitute at I362 (I362D). In contrast, the activity of alanine substituted mutant (I362A) was comparable to the wild-type ERα [27]. The hydrophobic amino acids of AF-2 surface are important for stabilizing the hydrogen bonds between LxxLL motif and the residues of K366 and E546 [27]. The result from the I362D mutant suggested that the irregular surface charge of this area disrupts the agonist-dependent coactivator recruitment to the AF-2. We have characterized the activity of the mERα I362D mutant, which is another E2-insensitive AF-2 mutant ERα having a mutation on the rigid region of AF-2 in contrast to the mutation of the flexible region of AF-2 (i.e., AF2ER) (Figure 1).

The agonist activity through mERα I362D was not observed as expected. However, it was surprising that the 3x VitERE-mediated transcription of mERα I362D was activated by a higher concentration (100 nM) of E2 or the synthetic estrogen, diethylstilbestrol (DES), despite no activation of this mutant ERα at lower concentrations (1 and 10 nM) of these chemicals [16]. In addition, we observed the reversed antagonist function of SERMs and SERDs through mERα I362D similar to what was observed with the AF2ER mutant. The agonistic function of the antagonist chemicals, as well as the 100 nM E2 and DES-mediated transcriptional activity was totally eliminated by the truncation of AB-domains of the mERα I362D mutant [16]. This result clearly suggested that the ligand-dependent mERα I362D transactivation function was solely derived from the AF-1 activity.

Experimentally, the activity of ERα AF-1 could be monitored by the LBD truncation from the ERα protein. An LBD truncated mutant, mERα339 is a ligand independent, constitutively active mutant of ERα, which activity is derived from the AF-1. Interestingly, extending the C-terminus of mERα339 by 45 amino acids (mERα384) eliminated the ligand independent transcription activity. The mERα384 mutant contains the LBD region up to helix 3, which includes the I362 residue. Surprisingly, the I362D mutated mERα384 (mERα384-I362D) restored the ligand independent transcription activity [16]. These observations suggest that the region around I362 of helix 3 is involved in blocking of AF-1 activity, which is reduced by the mutation of I362D. The mERα384 that we used in our experiments possesses 14 extra amino acids in the C-terminus which were derived from the expression plasmid. When we added a stop codon at the 385th amino acid position of mouse ERα (mERα384-stop), the activity of mERα384-stop was same as the activity of mERα339 (unpublished information). The computational prediction of protein secondary structure indicated that the extended 14 amino acids of our mERα384 help to maintain the structure of helix 3, in contrast with the mERα384-stop, which lost the helix-3 structure (unpublished information). Thus, we believe that the helix 3 of the LBD is involved in the attenuation of AF-1 activity of unliganded ERα. Additionally, we found that the transcriptional activity of AF2ER-I362D (triple mutated mERα) was more sensitive and potent mutant than the AF2ER mutant. Since, several antagonists that did not activate the AF2ER mutant activated the AF2ER-I362D mutant, and that activity was AF-1-dependent. These results would suggest that the helix-3-mediated AF-1 attenuation activity is disrupted by the I362D mutation. This may be an explanation for the reversed antagonist activity through mERα I362D mutant.

**Figure 3:**
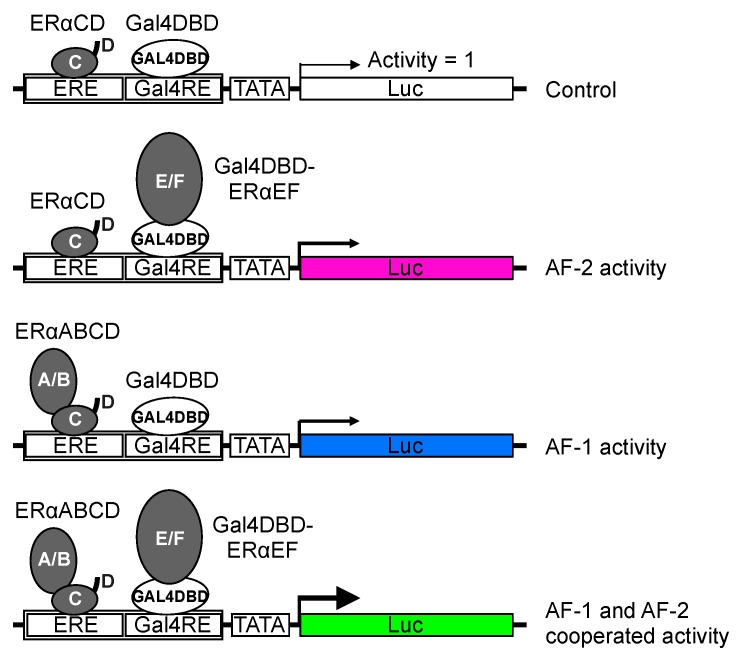
Schematic diagram of a hybrid reporter assay. The activity of reporter gene, which contains a Gal4-binding element (Gal4RE) juxtaposed to an ER binding element (ERE), coexpressed with ERα fragments was analyzed. In experiment, the activities were normalized by the activity of ERαCD and Gal4DBD expressing cells (Control; white). The cells that were coexpressed with ERαCD and Gal4DBD-ERαEF demonstrate AF-2 activity (pink). The cells that were coexpressed with ERαΑΒCD and Gal4DBD demonstrate AF-1 activity (blue). The cells that were coexpressed with ERαΑΒCD and Gal4DBD-ERαEF demonstrate AF-1 and AF-2 cooperative activity (green).

There are two possible mechanisms by which the C-terminal LBD can affect the N-terminal AF-1 activity. Specifically, the physical alteration of an ERα protein affects the association between LBD and AF-1 domain or the involvement of cellular factor(s) that may be bridging the LBD and AF-1 domain to control AF-1 activity. To test which mechanism is involved in the connection between LBD and AF-1 domain, we developed a novel method which used the expression plasmids for N-terminal and C-terminal ERα fragments to regulate the reporter gene transcription. The N-terminal ERα expression plasmid contains A through D domains (mERαABCD). The C-terminal ERα expression plasmid contains E and F domains fused with Gal4 DBD (Gal4DBD-mERαEF). The physically separated ERα expression plasmids were cotransfected with the luciferase reporter plasmid fused with an ERE and a Gal4 responsive element (Gal4RE) combined element (ERE-Gal4RE) (Figure 3). We found that antagonists did not enhance the N-terminal and C-terminal separated AF2ER-I362D-mediated transactivation function differently from the function of the full-length AF2ER-I362D mutant [16]. This result suggested that the physical alteration of an ERα protein is involved in the LBD-mediated AF-1 regulation, instead of the involvement of cellular bridging factors. Additionally, we found that the homo-dimerization activity of AF2ER-I362D-LBD was also induced by antagonists, coinciding with the transcription activity of AF2ER-I362D mutant [16]. It is likely that the prevention of coactivator interaction with the AF-2 allowed the ERα LBD dimerization to occur in a different manner than the dimerization of coactivator interacting ERα. This altered dimer structure may be involved in the exposing the N-terminal AF-1 domain for transcriptional activation.

The physical interaction between the LBD and the N-terminal A-domain of human ERα (1-37 amino acids of hERα) had been reported previously [41]. The report of Métvier et al. suggested that the A-domain interacts with the LxxLL motif binding cleft of LBD; the event is associated with the transcriptional inactivation of ligand unbound hERα. Our study showed that the LBD possesses an AF-1 inactivation domain and it works through LBD truncated mERα mutant as well, suggesting that the firm interaction between domains is not necessary for the AF-1 inactivation function. Taken together, these results suggest that the mild interaction of N-terminal structure (i.e., AB-domains) and the LBD may produce transcriptionally quiescent condition of a ligand-unbound ERα molecule. Dissociation of AB-domains from the LBD may be a key step of ligand-dependent ERα activation. The study of Kobayashi et al. suggested that the E2-bound hERα LBD interacts with AB-domains through p300 for transcriptional activation [42]. Recently, the structure of hERα dimer and coactivators (SRC-3 and p300) complex on the ERE was revealed by using the cryo-EM technique [43]. The structural model suggested that the E2-bound hERα dimer recruits two SRC-3s on the AF-2 domain of each hERα molecule and one p300 is associated with two SRC-3 molecules. In addition, the report suggested that the AF-1 domain is involved in the SRC-3 recruitment. When the agonists (e.g., E2) bind to the LBD, the cellular factors, such as SRC-3 and p300 may induce the AF-1 dissociation from the LBD and interact with AF-2, and as a consequence, the factors connect between AF-1 and AF-2 domains. On the other hand, when certain SERMs bind to the LBD, the ligand might induce AF-1 dissociation from the LBD without coactivator recruitment to the AF-2. The formation of LBD dimer with SERMs may be a cause of AB-domains (i.e., AF-1) dissociation from the LBD and the activation of AF-1-dependent transcription.

## 4. The F-Domain Functionality for the SERM-Dependent Partial Agonist Activity

The partial-agonist activity of SERMs and of 4OHT in particular has been discussed [44,45]. The partial-agonist activity of 4OHT for ERα is derived from the AF-1-mediated transcription activity [44,46]. However, the molecular mechanism of 4OHT-mediated ERα AF-1 activation is still unclear. We found a similarity between the partial-agonist activity of 4OHT for wild-type ERα and the antagonist-mediated AF2ER mutant activation. When we used the wild-type ERα-LBD/EF domains for a M2H assay, we found that 4OHT is totally unable to activate wild-type ERα-LBD-mediated transcription activity, similar to AF2ER-LBD. As we described above, this allowed us to perform a M2H assay for examining the 4OHT-dependent homo-dimerization activity of wild-type ERα-LBD [47]. The results of the M2H assay clearly suggested that the homo-dimerization of wild-type ERα-LBD can be induced by 4OHT. Interestingly, this LBD dimerization activity was strongly attenuated by truncating the F-domain, coinciding with the reduction of the 3x VitERE-mediated transcription activity [35]. These similar characteristics to the AF2ER mutant suggest that the mechanism of 4OHT-mediated activation of wild-type ERα AF-1 may be the same as AF2ER mutant. Principally, 4OHT induces homo-dimerization of ERα providing for enhancement of ERE binding and exposure of AF-1.

Our observations suggested that the F-domain is likely to be involved in the 4OHT-mediated wild-type ERα-LBD homo-dimerization. It is known that the ERα LBD structures using X-ray crystallography include only the E-domain but do not include the F-domain [48]. Attempts to resolve the F-domain structure by crystallography using the mouse ERα LBD (i.e., mERαEF) have unfortunately been unsuccessful. During these studies of the mERαEF protein expression process, we recognized significant proteolysis of the F-domain in the bacterial culture without ligand. Interestingly, this degradation was prevented by the addition to the bacterial culture medium of clomiphene, a SERM like chemical which has similar characteristics as 4OHT [49]. We also applied this procedure for the purification of human ERα LBD (i.e., hERαEF), however the F-domain proteolysis of hERαEF protein could not be blocked by addition of clomiphene (unpublished observation). The structure of the F-domain and its interactions with other domains of the ERα is still unknown. We presume however that the F-domain is involved and plays a role in the conformational stabilization of mERα LBD homo-dimer, at least when it binds with 4OHT or some other SERMs, such as clomiphene.

**Figure 4:**
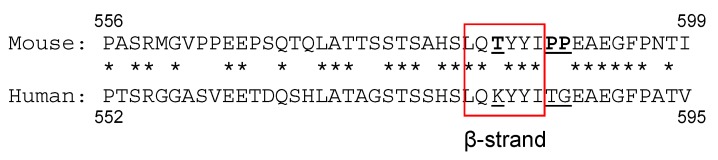
The amino acid sequence of mouse and human ERα F domains. The stars indicate identical amino acids between two species. The region of predicted β-strand is marked with red square. Mouse specific residues around the predicted β-strand are denoted as bold letters.

We noticed that the homology of F-domain between mouse and human ERα proteins is remarkably lower (75.6% similarity) than the homology to the entire ERα protein (94.7% similarity). The differential activities of estrogenic compounds in different species have been considered [50]. Due to the high overall homology of ERα protein among species, it has been thought that the differential metabolisms of chemicals may be the cause of differential estrogenic biological activities rather than species differences of ERα structure. A few studies considered that the structural difference of ERα between animals may contribute to the differential estrogenic activities [51]. Petit et al reported the differential functional amino acids in C-domain using the human-rainbow trout ERα chimera [52]. However, no research has focused on the functionality of the F-domain related to the species difference of ERα action. To assess the functionality of ERα F-domain, we generated chimeric ERα expression constructs having the F-domains of mouse and human receptors exchanged. We analyzed the transcription activity, coactivator interaction activity and LBD homo-dimerization activity of the mouse-human F-domain swapped ERα using cell based in vitro assays [53]. We found that the transcriptional activity of mouse wild-type ERα is more potent than human wild-type ERα under our experimental conditions. 4OHT-mediated differential transcription activities of mouse and human ERα were reversed by F-domain swapping, but not E2-mediated transcription activities. The structure of ERα F-domain is still not known, however, the predicted structures in the F-domain had been reported previously [54,55]. Further studies focusing on the predicted β-strand region in the F-domain revealed that this region is linked with the species difference of 4OHT-mediated ERα transcription activity [53]. Interestingly, there are several species-specific residues in this region (Figure 4). The substitution of three mouse specific residues into the human ERα (hERαTyyiPP) resulted in a similar level of 4OHT-mediated transcription activity as the mouse wild-type ERα, but did not alter E2-mediated transcription. Additionally, 4OHT-mediated LBD homo-dimerization activity of hERαTyyiPP-LBD was significantly greater than wild-type hERα-LBD [53]. These results implicate that the F-domain is highly involved in the 4OHT-mediated ERα transcription activity.

## 5. Conclusions

The transcriptional activity of ERα is regulated by various ligands. Explanation for the differential transcriptional activity of ERα ligands has focused on the activity of ligand-dependent coactivator recruitment to the AF-2. Especially, the importance of ligand-dependent positioning of helix12 for the efficacy of coactivator recruitment has been considered as an explanation for the partial/weak agonist activity of ligands [56,57]. On the contrary, the AF-1-mediated partial agonist activity of SERMs is not explained by the activity of coactivator recruitment to the AF-2. As we described here, the efficacy of SERM-dependent homo-dimer formation is important when considering the AF-1-mediated partial agonist activity of SERMs. This mechanism provides a possible developmental target for the generation of novel SERM chemicals to selectively control ERα AF-1-mediated physiological responses.

## 6. Summary

1. Helix 12 of ERα LBD defines ligands as agonists or antagonists. Antagonists can work as agonists through ERα mutants with a disrupted helix 12 and that transcriptional activity is derived from AF-1.
2. Helix 3 of ERα LBD contributes to the attenuation of AF-1 activity. Anomalous charge on helix 3 disrupts the LBD functions of AF-1 suppression and AF-2 transactivation.
3. SERMs induce ERα homo-dimerization through the LBD without recruitment of AF-2 coactivators. The AF-1-dependent transcriptional activity of SERMs correlates with the activity of LBD homo-dimerization.
4. The F-domain of ERα contributes to the SERM (4OHT)-dependent LBD homo-dimerization.

## Abbreviations

ERα	Estrogen receptor alpha
SERM	Selective estrogen receptor modulator
DBD	DNA binding domain
LBD	Ligand binding domain
KO	Knockout
4OHT	4-hydroxytamoxifen
SERD	Selective estrogen receptor degraders
E2	17β-estradiol
Vit	Vitellogenin
M2H	Mammalian two-hybrid
DES	Diethylstilbestrol
ERE	Estrogen responsive element
Gal4RE	Gal4 responsive element
